# Unilateral Stimulation of Subthalamic Nucleus Does Not Affect Inhibitory Control

**DOI:** 10.3389/fneur.2018.01149

**Published:** 2019-01-07

**Authors:** Christian Mancini, Nicola Modugno, Marco Santilli, Luigi Pavone, Giovanni Grillea, Roberta Morace, Giovanni Mirabella

**Affiliations:** ^1^Department of Anatomy, Histology, Forensic Medicine and Orthopedics, Sapienza University, Rome, Italy; ^2^IRCCS Neuromed, Pozzilli, Italy

**Keywords:** deep brain stimulation, Parkinson's disease, stop-signal task, goal directed action, reaching arm movement

## Abstract

Despite the relevance of inhibitory control in shaping our behavior its neural substrates are still hotly debated. In this regard, it has been suggested that inhibitory control relies upon a right-lateralized network which involves the right subthalamic nucleus (STN). To assess the role of STN, we took advantage of a relatively rare model, i.e., advanced Parkinson's patients who received unilateral deep-brain stimulation (DBS) of the STN either of the left (*n* = 10) or of the right (*n* = 10) hemisphere. We gave them a stop-signal reaching task, and we compared patients' performance in two experimental conditions, DBS-ON and DBS-OFF. In addition, we also tested 22 age-matched healthy participants. As expected, we found that inhibitory control is impaired in Parkinson's patients with respect to healthy participants. However, neither reactive nor proactive inhibition is improved when either the right or the left DBS is active. We interpreted these findings in light of the fact that previous studies, exploiting exactly the same task, have shown that only bilateral STN DBS restores a near-normal inhibitory control. Thus, although null results have to be interpreted with caution, our current findings confirm that the right STN does not play a key role in suppressing pending actions. However, on the ground of previous studies, it is very likely that this subcortical structure is part of the brain network subserving inhibition but to implement this executive function both subthalamic nuclei must be simultaneously active. Our findings are of significance to other researchers studying the effects of STN DBS on key executive functions, such as impulsivity and inhibition and they are also of clinical relevance for determining the therapeutic benefits of STN DBS as they suggest that, at least as far as inhibitory control is concerned, it is better to implant DBS bilaterally than unilaterally.

## Introduction

In the real world the course of events cannot be fully predicted. There are thus instances in which the value of a planned action might suddenly change during the time gap between the moment when an action is chosen and the moment when motor output is about to be generated. Under these circumstances, the pending action must be suppressed to avoid awful consequences, such as being struck by a truck suddenly appeared on the road when we are about to cross it.

Given the importance of inhibitory control in implementing adaptive and flexible behavioral strategies, it is not surprising that a wide number of cortical and subcortical structures are involved in action inhibition (1, 2). However, both the key nodes of the inhibitory network and what roles they play are unresolved and debated.

On the one hand, it has been suggested that inhibition depends critically on the interactions of two regions of the frontal cortex of the right hemisphere, i.e., the inferior frontal gyrus (IFG) and the pre-supplementary motor area (3). These two regions would implement inhibition via the projections to the right subthalamic nucleus (STN), which would then suppress the activity of the premotor cortex (4, 5) and the primary motor cortex (6, 7). On the other hand, other evidence indicates that the functioning of the inhibitory network is much more complex (1). First, it includes more regions, e.g., the striatum (8, 9) and the cerebellum (10). Second, there is evidence that at least some regions of the network are activated bilaterally, suggesting that the two hemispheres cooperate (8, 11–13). The STN is among those regions. Several studies have provided evidence that this subcortical nucleus is involved in inhibitory control (14, 15). However, while some studies suggest that a key role in the implementation of this executive function is played by the right subthalamus (16), others have shown that both STN have to be active in order to restore a near-normal reactive^1^ and proactive^2^ inhibitory control (12, 13).

However, as it has been shown that STN DBS can modulate cortical plasticity of the primary motor cortex (17, 18), a possible objection to these latter results is that the chronic bilateral stimulation of STN might have induced some form of brain plasticity, which altered the inhibitory network making indispensable the activation of both DBSs for improving inhibitory control. For instance, the simultaneous DBS of the two STN might indirectly allow a greater functional connection between the two motor cortices. Therefore, the aim of the present work was to assess whether this could be the case exploiting a relatively rare model, i.e., Parkinson's patients bearing unilateral STN DBS. We hypothesized that if the absence of the lateralization of inhibitory control was an artifact due to the bilateral stimulation, then unilateral STN DBS should not affect either reactive (13) or proactive inhibition (12). Differently, if the role of the right STN in this executive function was masked in our previous experiments, in the current one patients bearing a DBS on the right side should exhibit better inhibitory control when the stimulation is ON than when is OFF. In addition, patients bearing a DBS on the left side should not show any improvement of inhibition, independent of the DBS state.

## Materials and Methods

### Participants and Clinical Assessment

From the outpatients of the Parkinson's unit of the IRCCS Neuromed Hospital we selected 20 patients who had undergone unilateral STN DBS implantation to address poor control of cardinal PD symptoms, and/or side-effects induced by chronic administration of dopaminergic medicaments. Ten patients had the DBS in the right STN (two females; age range 54–72, mean ± SD, 60.6 ± 5.9 years) and 10 had the DBS in the left STN (five females; age range 50–71, mean ± SD, 58.6 ± 7.6 years). At our center, patients meeting criteria for STN DBS always undergo unilateral surgery during which a DBS macroelectrode (model 3389, Medtronic Ltd, Minneapolis, MN, USA) is inserted into the STN contralateral to the most affected body side. Subsequently, patients undergo the contralateral surgery when and if needed. Surgical procedures for DBS implantation have been reported in detail previously (13).

All patients were affected by idiopathic PD and they were in stable treatment with chronic STN stimulation complemented by administration of L-dopa and dopamine agonists for at least 6 months prior to study participation. Patients did not present severe sensory deficits, severe tremor, overt signs of dementia (mini-mental state examination, MMSE > 24) or any other neurological disease besides PD (as assessed by a standard neurological examination). All patients were right-handed as assessed by the Edinburgh handedness inventory and they were matched for the severity of the disease (see Table 1 for a summary of all clinical data). At the time of testing, no patient exhibited symptoms of impulse control disorder (19). A recent meta-analysis by Manza et al. (20) revealed that the effect of dopaminergic medication on response inhibition depends on disease duration. While inhibitory control seems to benefit from dopaminergic treatment in early-stage Parkinson's patients, it does not have an effect in the moderate-to-advanced stages of the disease. A plausible hypothesis to explain the diminished efficacy of dopaminergic drugs on inhibition in the moderate-to-advanced patients is that in those patients, too few dopaminergic cells for the drugs to operate on survived. This is very likely the condition of all our patients who underwent DBS implantation for treating advanced Parkinson's disease. Therefore, in order to minimize the risk of deteriorating motor ability of patients during the DBS OFF state, we tested all patients in ON-therapy, i.e., they were allowed to take their habitual doses of drugs. In order to have reference values for inhibitory control, we tested 22 right-handed healthy participants (12 females; age range 50–72, mean ± SD, 58.9 ± 6.8 years; years of education 12.9 ± 4.6) with normal or corrected-to-normal vision and without a history of neurological diseases. The ages of control participants and their education were not statistically significantly different from those of PD patients [one-way ANOVA on age, *F*_(2, 39)_ = 0.27; *p* = 0.77; one-way ANOVA on education, *F*_(2, 39)_ = 0.78; *p* = 0.46].

**Table 1:** Clinical data of Parkinson disease (PD) patients with deep brain stimulation (DBS) implant in the right and left subthalamic nuclei (STN) participating in the experiment.

**N**	**Education (years)**	**Age of onset**	**Years since diagnosis**	**Months since implantation**	**LEDD**	**UPDRS III**				**Hoehn and Yahr**	**MMSE**
						**MED on STIM on**	**MED on STIM off**	**MED off STIM on**	**MED off STIM off**		
**DBS Right STN**											
1	8	54	18	34	800	19	29	29	45	3	24.7
2	13	46	14	12	1200	16	23	18	36	3	24
3	8	48	11	97	600	5	35	12	48	3	29
4	13	52	16	9	900	24	30	31	44	2.5	25.4
5	8	50	12	6	850	12	21	31	43	2.5	24
6	18	38	16	8	500	9	19	13	32	2.5	29
7	13	32	23	26	540	15	24	25	38	3	30
8	18	35	19	9	1200	17	25	20	33	2.5	26
9	13	53	10	7	600	16	24	19	32	2.5	27
10	8	51	8	7	610	10	20	13	28	3	29.7
Mean	12.0	45.9	14.7	21.5	780.0	14.3	25	21.1	37.9	2.8	26.9
(SD)	3.9	8.0	4.6	28.1	258.7	5.46	4.99	7.48	6.76	0.3	2.4
**DBS Left STN**											
1	8	61	10	7	770	12	21	23	48	3	26.3
2	8	42	10	7	850	8	14	14	30	2.5	28
3	8	53	13	26	700	18	40	50	61	3	30
4	13	41	20	12	820	16	25	30	42	3	29.5
5	13	30	20	5	500	6	12	12	24	2.5	25.3
6	18	47	11	9	540	7	17	20	26	2.5	24
7	8	41	15	60	720	7	20	16	28	2.5	28.7
8	17	52	16	30	400	9	18	16	28	3	30
9	8	45	7	6	400	9	12	12	18	2.5	28
10	8	34	18	29	850	10	14	29	36	3	24.9
Mean	10.9	44.6	14.0	19.1	655.0	10.2	19.3	22.2	34.1	2.8	27.5
(SD)	4.0	9.2	4.5	17.5	179.5	4.0	8.4	11.7	12.9	0.3	2.2

*For each patient sex, age, years of education, age of PD onset, years since diagnosis, months since surgery, post-operative levodopa equivalent daily dose (LEDD), Unified Parkinson's Disease Rating Scale (UPDRS) motor scale (III) in each DBS condition (STIM on/off), and in each medication condition (MED on/off), Hoehn and Yahr scores (measured in DBS OFF and after about 12 h of levodopa deprivation), and post-operative mini mental state examination (MMSE) scores are given. Neither the years since diagnosis [t-test t_(18)_ = 0.34; p = 0.7], nor the months since implantation [t-test t_(18)_ = 0.23; p = 0.8], nor the post-operative LEDD [t-test t_(18)_ = 1.25; p = 0.2], nor the UPDRS III score in any of the four conditions [all t-tests p > 0.05], nor the Hoehn and Yahr score [t-test t_(18)_ = 0; p = 1], nor the MMSE score [t-test t_(18)_ = −0.57; p = 0.6] were significantly different between right and left STN DBS patients (for the statistics on age and education see the main text)*.

All participants gave their informed consent and they knew they could withdraw from the study at any time. All the procedures were approved by the Institutional Ethics Committee of IRCCS Neuromed and were performed in accordance with the ethical standards of the Declaration of Helsinki of 1964.

### Post-surgical Reconstruction of the Position of DBS Contacts

To verify the placement of the active DBS contact (DBS electrode had four platinum–iridium cylindrical surfaces with a diameter of 1.27 mm and an edge-to-edge spacing of 0.5 mm) with respect to STN we followed the following procedure.

**Figure 1:**
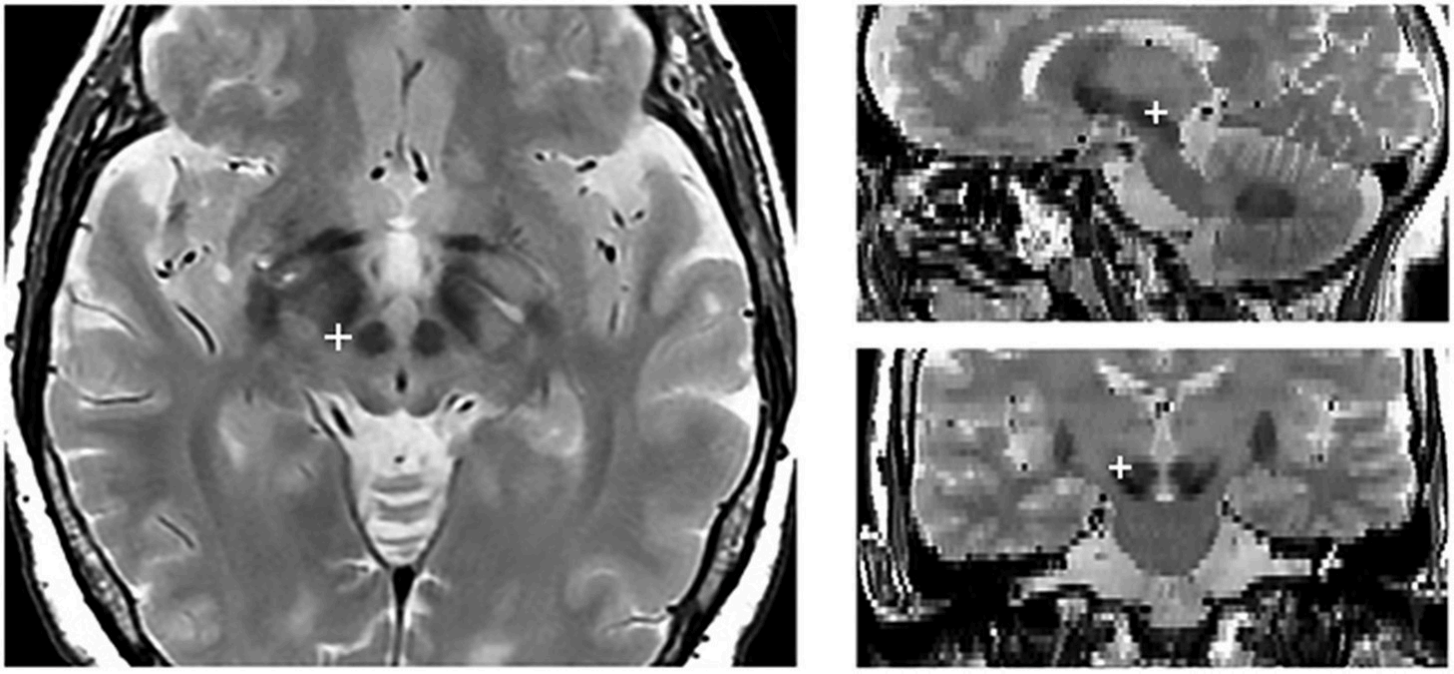
Localization of the active electrode contact in an example subject. The red cross indicates the position of the active contact in the axial (right), sagittal (upper left), and coronal (lower right).

Each patient underwent both a preoperative 3-dimensional fast spoiled gradient echo T1-weighted 3.0 T MRI scan (slice thickness 1 mm, repetition time 7 ms, flip angle 13°, matrix size 256 × 256) to identify brain structures and a T2-weighted 3.0 T MRI scan (slice thickness 2 mm, repetition time 7,560 ms, flip angle 90°, matrix size 480 × 256) to show STN position. About a month after the surgery, a CT scan was acquired on a CT LightSpeed Pro (slice thickness 0.625 mm, exposure time 1,000 ms, kVp 120, matrix size 512 × 512). Finally, the two preoperative MRI scans were merged with the post-operative CT scan using Optivise software (Medtronic Inc.). The locations and the stimulation parameters of the active electrode contacts are reported in Table 2, while Figure 1 illustrates the reconstruction of the position of the active DBS contact for an example subject.

**Table 2:** Localization of the active contact/contacts of the DBS electrode across Parkinson's patients.

**n**	**Active Electrodes**	**Amplitude (mA)**	**Impedance (Ω)**	**Pulse (μ s)**	**Frequency (Hz)**
**DBS RIGHT**					
1	Zi^+^, STNd^−^ (*)	2.42	1239	60	140
2	STNv	2.48	1936	60	130
3	STNd	2.45	1426	60	210
4	STNd	3.26	1012	60	180
5	STNv	2.53	989	60	180
6	Zi	2.50	1198	60	190
7	Zi	2.22	1486	60	185
8	Not localized	1.96	1023	60	180
9	Zi	2.80	1248	60	130
10	Zi	2.20	1137	60	130
**DBS LEFT**					
1	Zi	1.62	1236	60	180
2	Zi	2.27	1101	60	160
3	STNd	2.65	869	60	130
4	STNd	2.30	1002	60	180
5	STNd^+^, Zi^−^ (*)	2.05	1364	60	180
6	STNd^+^ STNd^−^ (*)	2.13	1879	60	180
7	Not localized	2.44	1025	60	185
8	STNd	2.67	1311	60	190
9	STNd	2.09	1098	60	180
10	STNd^+^, STNd^−^ (*)	2.69	1116	60	180

For each patient the position of active contact/contacts, amplitude, impedance, pulse and frequency of stimulation are shown. Active electrodes indicated by a star (*) have a bipolar configuration, with cathode (+) and anode (−). Two patients could not be localized due to technical problems. Neither the amplitude [t-test t_(18)_ = 1.23; p = 0.23], nor the frequency of stimulation [t-test t_(14.5)_ = 0.82; p = 0.42] were significantly different between right and left STN DBS patients. STNd, dorsal subthalamic nucleus; STNv, ventral subthalamic nucleus; Zi, zona incerta.

#### Experimental Paradigms

Experimental procedures have been already described in detail previously (21, 22). Participants were seated in a darkened and silent room, in front of a 21″ PC monitor (refresh rate 60 Hz, 640 × 480 resolution) on which visual stimuli were presented. Stimuli consisted of red circles (2.434 cd/m^2^) of 2.5 degrees of visual angle of diameter against a dark background of uniform luminance (<0.01 cd/m^2^). The PC was coupled with a touchscreen (MicroTouch; sampling rate 200 Hz) for touch-position monitoring. We positioned the chair so that their eyes were about 40 cm away from the PC monitor and patients could comfortably reach the stimuli projected on the screen with the right arm. The reaching movement involved distal and proximal muscles but not axial muscles.

Both patients and age-matched controls performed two tasks using their right (dominant) arm. First, they performed a go-only task and then a reaching version of the countermanding task. The go-only task was a simple reaction time (RT) task, aimed to measure RTs and movement times (MTs) of reaching arm movements. In go-only trials, participants had to reach and hold a central stimulus until it disappeared and, simultaneously, a peripheral target appeared 18.6 degrees of visual angle to the right (go-signal). Participants were instructed to reach the target as quickly as possible and to hold it for 300–400 ms.

**Figure 2:**
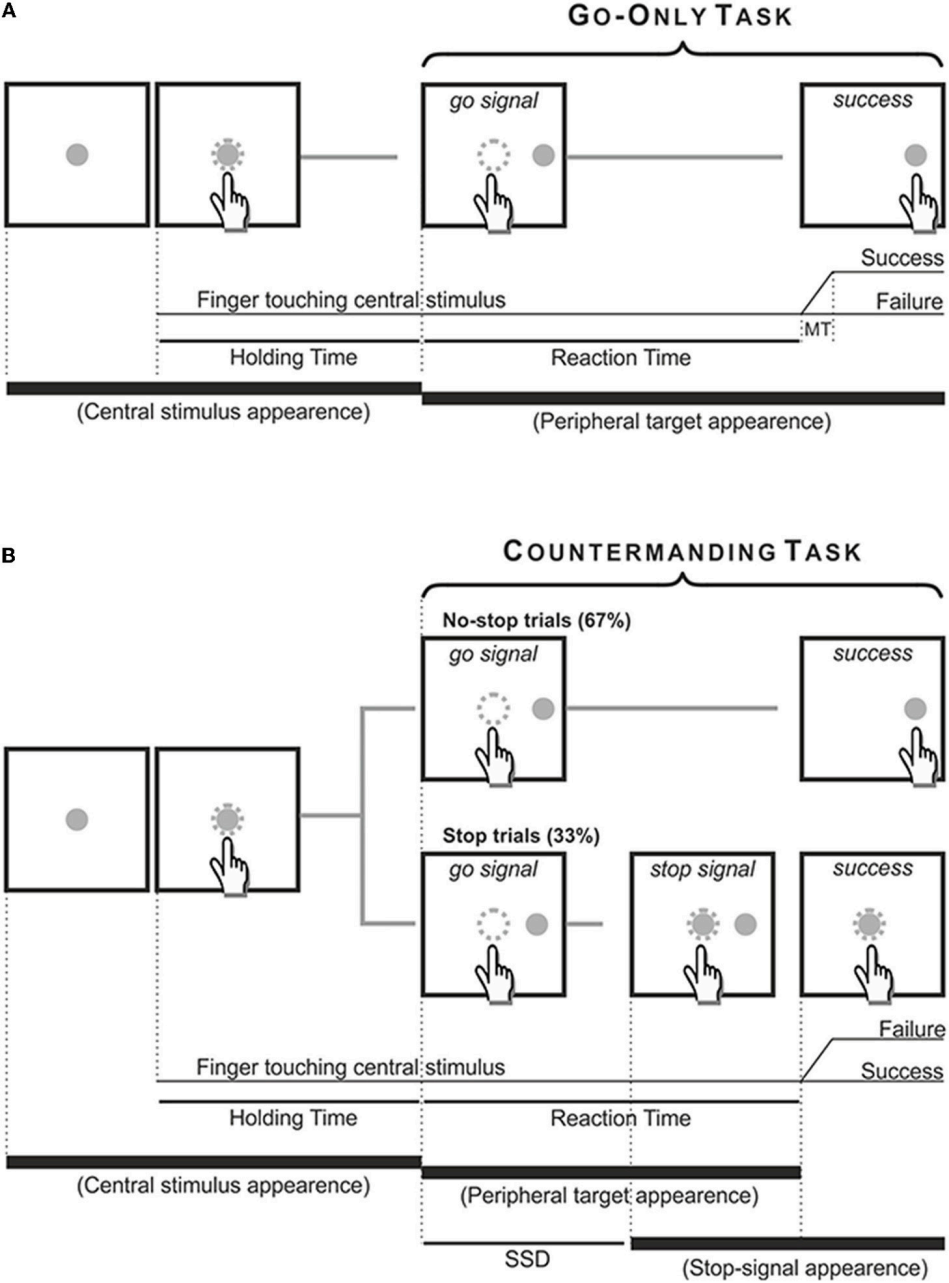
Temporal sequence of the visual displays for go-only, no-stop **(A)** and stop trials **(B)**. All trials began with the appearance of a central stimulus. The subject had to reach and hold it with the index of the right arm for 500–800 ms. **(A)** In the go-only task and in the no-stop trials of the countermanding task the central stimulus disappeared and, simultaneously, a target appeared 13.5 cm or 18.6 dva to the right, acting as a go-signal. Subjects were instructed to perform a speeded reaching movement toward the peripheral target and to hold it for 300–400 ms. Randomly, in 33% of trials of the countermanding task [stop trials, **(B)**] the central stimulus (stop-signal) reappeared at a variable delay after the go signal (stop signal delay, SSD), indicating that the subject should cancel the pending movement. In these stop trials, if subjects countermanded the planned movement keeping the arm on the central stimulus for a period of 400–600 ms, the trial was scored as a stop-success trial. Otherwise, if subjects executed the reaching movement the trial was scored as a stop-failure trial. The dotted circle (which was not visible to the subjects) indicates the size of the tolerance window for the touches (3.5 cm or 5 dva of diameter).

The countermanding task consisted of a pseudorandom mix of no-stop trials (67%) and stop trials (33%, Figure 2). No-stop trials were the same as go-only trials. In contrast, in stop trials the central stimulus, which represented the stop-signal, reappeared at a variable delay after the go-signal. In this instance, participants were instructed to suppress the pre-planned movements toward the peripheral target, holding the central stimulus for 400–600 ms. Trials in which participants successfully withheld the movement were defined stop-success, while those in which they could not refrain from moving were defined stop-failure. Auditory feedback was given for correct responses.

The stop-signal delay (SSD) is a critical dependent variable because stopping becomes increasingly difficult with its lengthening. The SSD was changed using a staircase procedure (23) with a 50% performance criterion. If participants succeeded in stopping the response, then the SSD increased by 39.9 ms (three refresh rates); otherwise, the SSD decreased by the same amount of time. The starting value of the SSD was 119.7 ms (nine refresh rates). We verbally informed participants that the probability of stopping would approximate to 50%, irrespective of whether they were postponing their response (a common strategy to make inhibition on stop trials easier). In addition, we set a maximum RT for no-stop trials, i.e., whenever the RTs were >800 ms, no-stop trials were aborted. However, those trials were kept for the final analysis to avoid cutting the right tail of the RT distribution, and they accounted for 1.4–6% of the total no-stop trials in patients and controls, respectively.

Parkinson's patients performed the tasks in two experimental conditions: (a) DBS-OFF; (b) DBS-ON. All tests were performed 60 min after the DBS was switched off or on so that they were tested in near-steady motor status (24). Stimulation conditions were counterbalanced across patients and administered in one experimental session.

In each condition, patients were required to complete three or four blocks of 120 countermanding trials (360–480 trials) and about 90 go-only trials. Overall, each patient performed 900–1,140 trials. Resting periods were allowed between blocks whenever requested. Before starting the task, about 50 or 60 practice trials were given for familiarizing participants with the apparatus. Age-matched healthy participants were required to complete four blocks of 108–120 countermanding trials (432–480 trials) and about 90 go-only trials.

### Data Analyses

As behavioral parameters, we considered RTs, MTs and the stop-signal reaction times (SSRTs). RTs were computed as the difference between the time of the go-signal presentation and the onset of movement. MTs were determined as the difference between the time of movement onset and the time at which the peripheral target was touched. Trials with RTs shorter/longer than the mean minus/plus three SDs were excluded from the analysis. Overall 1.8 and 0.4% of the data were discarded in patients and controls, respectively. The SSRT represents the estimate of stop latency (25), and it was estimated exploiting the integration method, which gives the best estimate when proactive slowing occurs (26). The SSRT provides a measure of reactive inhibition, i.e., the ability of a subject to react outright to the stop signal. In contrast, proactive inhibition, i.e., the ability of subjects to shape their response strategy in anticipation of known task demands as the awareness of the fact that sometimes a stop-signal could have been presented, was assessed by comparing the RTs and the MTs of no-stop trials vs. those of go-only trials. In fact, it has been shown that when the subject executes a no-stop trial, its RT is lengthened and its MT is shortened with respect to situations in which the same movement has to be performed in the context of the go-only trial. This “context effect” (27) represents an optimization of costs and benefits because longer RTs are compensated by shorter MTs and vice versa.

Different types of analysis of variance (ANOVA) were used depending on the experimental design in order to assess changes in RTs, MTs, and SSRTs. Bonferroni corrections were applied for all multiple comparisons. To contrast cumulative distributions of RTs and of MTs obtained in no-stop and go-only trials, two-sample Kolmogorov–Smirnov tests were used. χ^2^-tests were used to determine whether there were significant differences between the occurrences of the context effects.

We measured the “effect-size” by computing the partial eta-squared ($\eta_{\text{p}}^{2}$) for each ANOVA (with values of 0.01, 0.058, and 0.139 indicating small, medium and large effects, respectively), and Cohen's d for *t*-tests (with values of 0.2, 0.5, and 0.8 indicating small, medium, and large effects) (28). Finally, to quantify the strength of null hypotheses, we calculated the Bayes factors (BF10) with an r-scale of 0.707 (29). BF10 values < 0.1 and < 0.33 provide strong and moderate support, respectively, for a null hypothesis compared to the alternative hypothesis. For the sake of clarity, and to improve readability, we report just significant results, unless otherwise indicated. Data will be freely available from the Open Science Framework platform (https://osf.io/pfeqv/).

## Results

First of all, we assessed whether the staircase algorithm worked equally well for patients and controls. To this end, we compared the average proportion of trials in which participants moved the arm despite the stop signal [P (failure); see Table 3] using a one-way ANOVA (levels: DBS ON-right, DBS ON-left, and Controls). As we did not found a main effect [*F*_(2, 39)_ = 0.35, *p* = 0.28; $\eta_{\text{p}}^{2}$ = 0.02; BF_10_ = 0.25] we concluded that the staircase algorithm worked similarly well for all groups. Second, we checked whether a basic assumption of the race model, i.e., the stochastic independence between the go and the stop process, was fulfilled (25). Reaching movements were produced in both the no-stop trials and the stop-failure trials but, according to the model, the latter was initiated because the go process finished before the stop process. Therefore, stop-failure trials would be expected to have a shorter RT than no-stop trials. This was the case, as shown in Table 3. A two-way mixed-design ANOVA [between-subjects factor: Group (right-DBS ON, left-DBS ON, Controls); within-repeated-subject factor: Trial type (RT no-stop trials, RT stop-failure trials)] showed that stop-failure trials were faster than no-stop trials [*F*_(2, 39)_ = 459.6, *p* < 0.0001; $\eta_{\text{p}}^{2}$ = 0.92; BF_10_ = 786.1]. No other significant effects were found. In addition, for each healthy subject and patient, via a two-sample Kolmogorov–Smirnov test we tested whether the individual distributions of the RTs of stop-failure trials were different from those of no-stop trials. In all subjects, we found that the former trials were faster than the latter (all *p* < 0.05). Overall, these results indicate that the data allowed us to compute a reliable estimate of the SSRT.

**Table 3:** Summary of behavioral values of arm movements for Parkinson's disease patients with deep brain stimulator (DBS) implanted on the right side (right-DBS), on the left side (left-DBS) in ON and OFF states, and for age-matched controls during the countermanding and the go-only tasks.

	**Right-DBS ON**	**Left-DBS ON**	**Right-DBS OFF**	**Left-DBS OFF**	**Controls**
Mean SSD	171.8 ± 60.7	253 ± 164.1	184.7 ± 90.1	238.8 ± 154.4	283.8 ± 110.5
P (failure)	0.51 ± 0.03	0.5 ± 0.03	0.53 ± 0.07	0.52 ± 0.04	0.50 ± 0.03
SSRT	259.2 ± 26.7	253.2 ± 35.5	258.1 ± 37.7	253.8 ± 36.1	221.4 ± 27.2
RT no-stop trials	468.9 ± 68.5	523.2 ± 135.2	460.2 ± 96.8	497.5 ± 123	526.9 ± 105.7
RT stop-failure trials	347.9 ± 35.6	432.7 ± 126.8	364 ± 68.3	404.9 ± 114.4	425.3 ± 99.9
RT go only trials	295.4 ± 29.6	290.5 ± 34.7	286.7 ± 51.5	279.5 ± 34.3	326.1 ± 113.1
MT no-stop trials	538.4 ± 130	538.4 ± 148.7	536.4 ± 120.2	593.8 ± 143.5	434.3 ± 105.2
MT go only trials	558.4 ± 158.9	566.8 ± 143.8	572.9 ± 176.7	585.5 ± 113.8	506.8 ± 128.9
Task Performance accuracy go only trials	0.88 ± 0.07	0.93 ± 0.05	0.83 ± 0.08	0.89 ± 0.1	0.90 ± 0.08
Task Performance accuracy no-stop trials	0.89 ± 0.05	0.9 ± 0.06	0.88 ± 0.06	0.89 ± 0.05	0.88 ± 0.09

*Task performance accuracy was defined as the ratio between the number of trials correctly executed and the total number of trials delivered, given by the sum of trials correctly executed, trials in which participants missed the target, trials in participants remained still on the central stimulus for more than 2 s, and trials in which they did not hold the central stimulus or the peripheral target for the requested amount of time. In all cases the average value across the samples (±SD) is reported*.

### Reactive Inhibition

**Figure 3:**
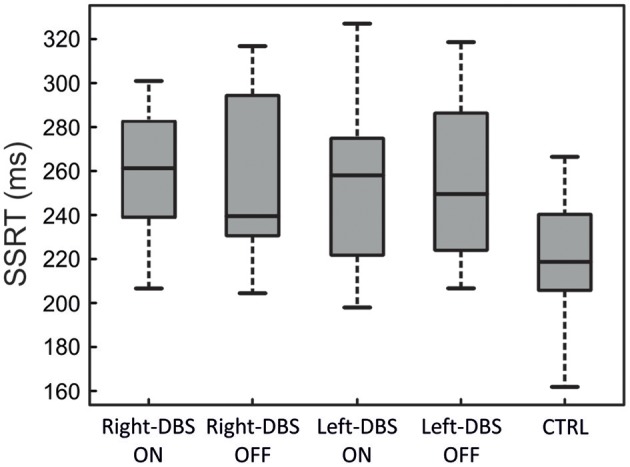
SSRT in right-DBS-ON and -OFF patients (*n* = 10), left-DBS-ON and -OFF patients (*n* = 10), and age-matched controls (*n* = 22). In the box plots, the boundary of the box closest to zero indicates the first quartile, a black thick line within the box marks the median, and the boundary of the box farthest from zero indicates the third quartile. Whiskers indicate values 1.5 times the interquartile range below the first quartile and above the third quartile.

Figure 3 and Table 3 illustrate one of two main findings of our work, i.e., the SSRT did not differ in patients either according to the DBS state or according to the side of DBS placement. However, reactive inhibition was impaired in patients, as the SSRT was longer than in healthy subjects. To compare the SSRT in patients, we ran a two-way ANOVA with mixed design (between-subjects factor: Group [right-DBS, left-DBS]; within-subjects-repeated factor: DBS-state [DBS-ON, DBS-OFF]). We did not find any significant results (see Table 4). The effect size estimate, together with the values of the BF_10_, support the conclusion that inhibitory control of right-DBS and left-DBS patients do not show significant differences, irrespective of the DBS state.

**Table 4:** Results of the statistical analysis of the mean SSRTs across right-DBS patients, left-DBS patients, and age-matched controls.

	**Value of parameters**	***p*-values**	**Effect size**	**BF_**10**_**
**Two way ANOVA: Group (right-DBS, left-DBS); DBS-state: (DBS-ON, DBS-OFF)**				
Main effect: Group	*F*_(1, 18)_ = 0.12	*p* = 0.73	$\eta_{\text{p}}^{2}$ = 0.007	0.34
Main effect: DBS state	*F*_(1, 18)_ = 0.002	*p* = 0.96	$\eta_{\text{p}}^{2}$ = 0.000	0.31
Interaction: Group*DBS state	*F*_(1, 18)_ = 0.12	*p* = 0.89	$\eta_{\text{p}}^{2}$ = 0.001	0.47
**One way ANOVA: Group (right-DBS-ON, left-DBS-ON, Controls)**				
Main effect: Group	*F*_(2, 39)_ = 7.11	***p =*** **0.002**	$\eta_{\text{p}}^{2}$ = 0.27	20.6
*post-hoc* test: R-ON vs. Controls	*t*_(18)_ = 3.6	***p =*** **0.006**	*d* = 1.44	27.6
*post-hoc* test: L-ON vs. Controls	*t*_(18)_ = 2.71	***p =*** **0.026**	*d* = 1.09	4.78
*post-hoc* test: R-ON vs. L-ON	*t*_(18)_ = 0.41	*p* = 1	*d* = 0.2	0.42

*Post-hoc tests (pairwise comparisons) had an adjusted alpha level corrected according to Bonferroni. The suffix “DBS-ON” indicates that the DBS was in the ON state. Statistically significant results are reported in bold. Bayes Factors report the ratio between the null vs. the alternative hypothesis (BF_10_); Partial eta-squared ($\eta_{p}^{\text{2}}$); Cohen's d (d). Note that BF_10_ represents continuous evidence, therefore it should not be interpreted as dichotomous threshold, such that a BF_10_ slightly larger than 0.33 still indicate a moderate evidence in favor of the null hypothesis. Bold text indicates statistically significant p-values*.

As there were no differences between the two DBS states, in the next step we compared the SSRTs of healthy subjects with those of right- and left-DBS in ON state, via a one-way ANOVA. We found a main effect (see Table 4) and *post-hoc* tests revealed that, as expected, controls had significantly better reactive inhibitory control than either right- or left-DBS patients. In contrast, the SSRT of right-DBS and left-DBS patients did not differ. Overall, these findings indicate that reactive inhibition is equally impaired in right- and left-DBS patients with respect to healthy participants.

### Proactive Control

We assessed proactive inhibitory control by measuring the context effect following three approaches.

**Figure 4:**
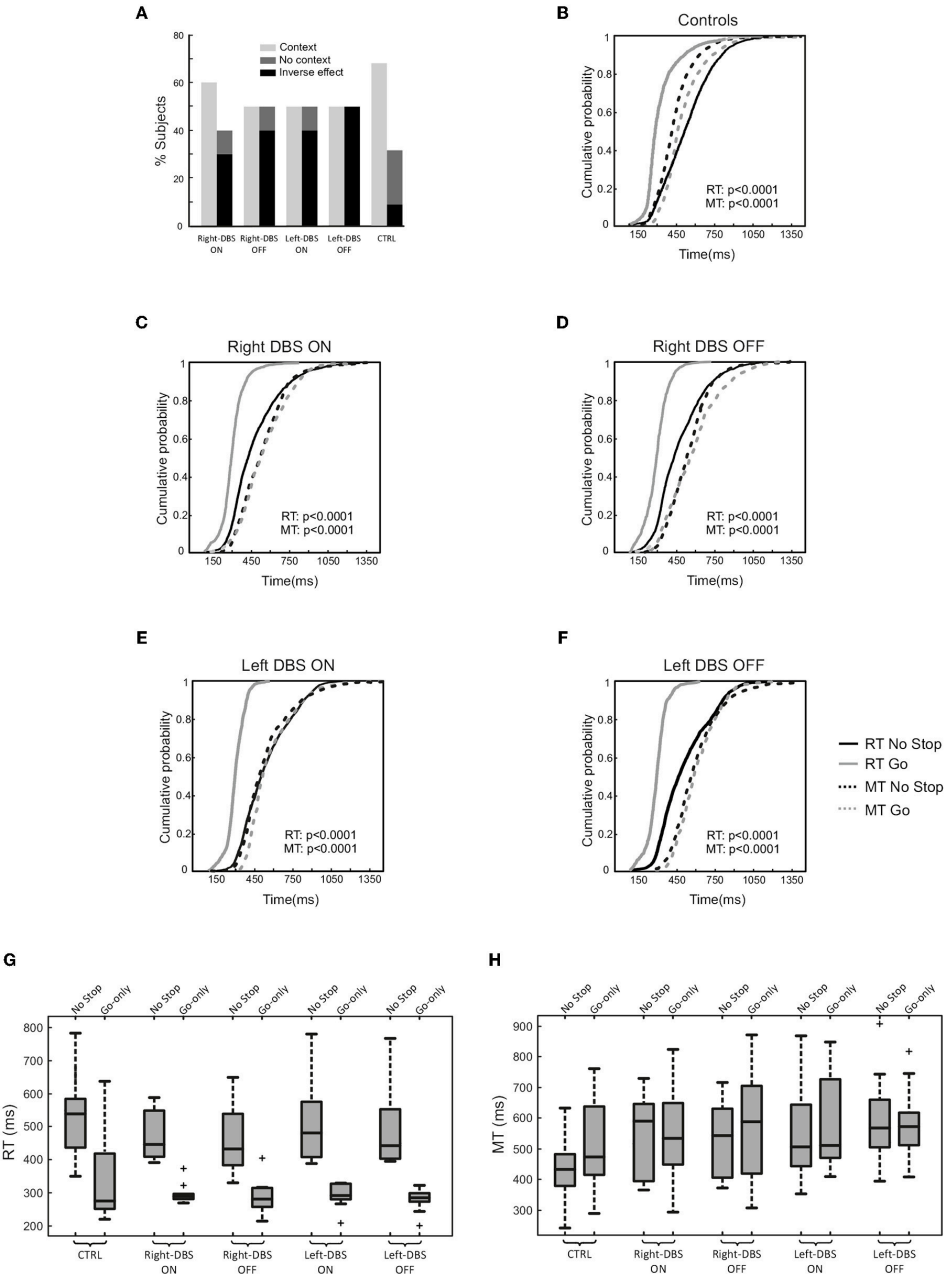
Proactive inhibitory control in age-matched controls (*n* = 22), right-DBS-ON and -OFF patients (*n* = 10), left-DBS-ON and -OFF patients (*n* = 10). **(A)** Percentage of right-DBS-ON and –OFF, left-DBS-ON and -OFF, and age-matched controls showing either (i) the context effect, i.e., the simultaneous significant increase in reaction times (RTs) and significant decrease in movement times (MTs) in no-stop trials with respect to go-only trials, (ii) the absence of a context effect due to a lengthening of both RTs and MTs in no-stop trials with respect to go-only trials (“reversed context”) or to a significant increase in RTs in no-stop trials with respect to go-only trials, but MTs of no-stop trials were not different from those of go-only trials (“no context”). **(B)** Cumulative distributions of RTs (solid lines) and MTs (dotted lines) of age-matched controls for go-only trials (gray) and no-stop trials (black). These cumulative distributions were obtained by collapsing together the cumulative distributions of RTs and MTs of no-stop and of go-only trials of single subjects. The *p*-value of the two-sample-Kolmogorov–Smirnov test is reported. **(C)** Same representation as in **(B)** for right-DBS patients in ON, and **(D)** for right-DBS patients in OFF state. **(E)** Same representation as in (B) for left-DBS patients in ON, and **(F)** for left-DBS patients in OFF state. **(G)** Box plot of RTs, and **(H)** of MTs of no-stop and go-only trials in right-DBS-ON, right-DBS-OFF, left-DBS-ON, left-DBS-OFF patients and age-matched controls. Outliers are represented by crosses, other conventions as in Figure 3.

First, we assessed the occurrence of the context effect in each participant (within-subject approach). With this aim, we considered whether the individual distributions of RTs and MTs of no-stop and go-only trials were significantly different via the two-sample Kolmogorov–Smirnov test. Thereafter, we computed the percentage of subjects who showed a context effect, namely a simultaneous increase in RTs and decrease in MTs in no-stop trials with respect to go-only trials. As shown in Figure 4A, we found that while 68.2% of controls had a context effect, the percentage of occurrences in DBS patients was lower. In fact, right- and left-DBS patients in the OFF state, as well as left-DBS patients in the ON state, showed the context effect in only 50% of instances, a value significantly smaller than that of controls [$\chi_{\left(1\right)}^{2}$ = 6.69, *p* = 0.01]. Right-DBS patients in the ON state had a slightly higher frequency of context effects, i.e., 60%, a value which was not significantly different from that of controls [$\chi_{\left(1\right)}^{2}$ = 1.39, *p* = 0.24]. This was possibly due to the random variability of the relatively small sample of patients.

Second, we created cumulative distributions of RTs and of MTs of go-only vs. no-stop trials by combining data from single participants (population approach, Figures 4B–F). As expected, healthy subjects had longer RTs and shorter MTs in no-stop trials than in go-only (11, 12, 27). Even though the overall effect on MTs was much smaller in DBS patients, the MTs of go-only trials seemed to be still longer than those of no-stop trials (Figures 4C–F; Kolmogorov–Smirnov test, all *p* < 0.0001). However, it should be stressed that the Kolmogorov-Smirnov test is sensitive to subtle shifts in the shapes of two distributions. Therefore, when the distributions are very close to each other this approach has a limitation as it might not reveal an overall shift in distributions but their different shapes. Assuming that this interpretation is the right one, we might assert that patients did not show a context effect irrespective of the DBS state.

Third, we compared the means of RTs and MTs of no-stop trials and go-only trials in the two groups of patients (Figures 4G–H) via two three-way ANOVAs with mixed design (between-subjects factor: Group [right-DBS and left-DBS]; within-subjects-repeated factor: Trial type [RT/MT no-stop trials, RT/MT go-only trials] and DBS state [DBS-ON, DBS-OFF]). As reported in Table 5, we found that RTs of no-stop trials were significantly longer than those of go-only trials. No other effects were found. Finally, as the previous analyses never showed differences between the two DBS states, we compared the RTs and the MTs of no-stop trials and go-only trials of healthy subjects with those of right- and left-DBS in the ON state, via two two-way ANOVAs with mixed design (between-subjects factor: Group [right-DBS, left-DBS, and controls]; within-subjects-repeated factor: Trial type [RT/MT no-stop trials, RT/MT go-only trials]). We found that RTs of no-stop trials were significantly longer than those of go-only trials, while MTs showed the opposite pattern, i.e., they were shorter in no-stop trials than in go-only trials (see Table 5).

**Table 5:** Results of the statistical analysis of mean RTs and MTs across right-DBS patients, left-DBS patients, and age-matched controls.

	**Value of parameters**	***p*-values**	**Effect size**	**BF_**10**_**
**Three-way ANOVA (factors: Group [right-DBS and left-DBS]; Trial type [RT no-stop trials, RT go-only trials] and DBS state [DBS-ON, DBS-OFF])**				
Main effect: Group	*F*_(1, 18)_ = 0.63	*p* = 0.44	$\eta_{\text{p}}^{2}$ = 0.034	0.28
Main effect: DBS state	*F*_(1, 18)_ = 1.56	*p* = 0.23	$\eta_{\text{p}}^{2}$ = 0.08	0.25
Main effect: Trial Type	*F*_(1, 18)_ = 63.8	***p <*** **0.0001**	$\eta_{\text{p}}^{2}$ = 0.78	3.1*10^13^
Interaction: Group*DBS state	*F*_(1, 18)_ = 0.2	*p* = 0.66	$\eta_{\text{p}}^{2}$ = 0.011	0.32
Interaction: Group*Trial Type	*F*_(1, 18)_ = 1.08	*p* = 0.31	$\eta_{\text{p}}^{2}$ = 0.06	0.64
Interaction: Trial Type*DBS state	*F*_(1, 18)_ = 0.12	*p* = 0.74	$\eta_{\text{p}}^{2}$ = 0.006	0.32
Interaction: Trial Type*DBS state*Group	*F*_(1, 18)_ = 0.12	*p* = 0.74	$\eta_{\text{p}}^{2}$ = 0.007	0.43
**Three-way ANOVA (factors: Group [right-DBS and left-DBS]; Trial type [MT no-stop trials, MT go-only trials] and DBS state [DBS-ON, DBS-OFF])**				
Main effect: Group	*F*_(1, 18)_ = 0.11	*p =* 0.75	$\eta_{\text{p}}^{2}$ = 0.006	0.27
Main effect: DBS state	*F*_(1, 18)_ = 1.48	*p =* 0.24	$\eta_{\text{p}}^{2}$ = 0.076	0.28
Main effect: Trial Type	*F*_(1, 18)_ = 1.15	*p =* 0.29	$\eta_{\text{p}}^{2}$ = 0.06	0.27
Interaction: Group*DBS state	*F*_(1, 18)_ = 0.75	*p =* 0.39	$\eta_{\text{p}}^{2}$ = 0.04	0.35
Interaction: Group*Trial Type	*F*_(1, 18)_ = 0.26	*p =* 0.62	$\eta_{\text{p}}^{2}$ = 0.014	0.30
Interaction: Trial Type*DBS state	*F*_(1, 18)_ = 0.88	*p =* 0.77	$\eta_{\text{p}}^{2}$ = 0.005	0.30
Interaction: Trial Type*DBS state*Group	*F*_(1, 18)_ = 0.62	*p =* 0.44	$\eta_{\text{p}}^{2}$ = 0.03	0.46
**Two-way ANOVA (factors: Group [right-DBS, left-DBS and Controls]; Trial type [RT no-stop trials, RT go-only trials])**				
Main effect: Group	*F*_(2, 39)_ = 1.01	*p =* 0.37	$\eta_{\text{p}}^{2}$ = 0.049	0.2
Main effect: Trial Type	*F*_(1, 39)_ = 130.6	***p <*** **0.0001**	$\eta_{\text{p}}^{2}$ = 0.77	4.5*10^11^
Interaction: Group*Trial Type	*F*_(2, 39)_ = 0.77	*p =* 0.47	$\eta_{\text{p}}^{2}$ = 0.038	0.26
**Two-way ANOVA (Group [right-DBS, left-DBS and Controls]; Trial type [MT no-stop trials, MT go-only trials])**				
Main effect: Group	*F*_(2, 39)_ = 2.14	*p =* 0.13	$\eta_{\text{p}}^{2}$ = 0.099	0.73
Main effect: Trial Type	*F*(1, 39) = 5.47	***p =*** **0.024**	$\eta_{\text{p}}^{2}$ = 0.123	2.02
Interaction: Group*Trial Type	*F*_(2, 39)_ = 1.14	*p =* 0.33	$\eta_{\text{p}}^{2}$ = 0.055	0.25

*Statistically significant results are reported in bold. Bayes Factors report the ratio between the null vs. the alternative hypothesis (BF_10_); Partial eta-squared ($\eta_{p}^{\text{2}}$); Cohen's d (d); The suffix “DBS-ON”/“DBS-OFF” indicates that the DBS was in the ON/OFF state. Bold text indicates statistically significant p-values*.

In order to check whether the faster responses in the no-stop trials induced a higher number of errors and vice versa in go-only trials (speed–accuracy tradeoff phenomenon) (30) we first compared the accuracy (see Table 3) in the two groups of patients via two three-way ANOVAs with mixed design (between-subjects factor: Group [right-DBS and left-DBS]; within-subjects-repeated factor: Trial type [% correct no-stop trials, % correct go-only trials] and DBS state [DBS-ON, DBS-OFF]). None of the factors showed statistically significant differences (Table 6). Therefore, we compared the accuracy of no-stop trials and go-only trials of healthy subjects with those of right- and left-DBS in the ON state, via two two-way ANOVAs with mixed design (between-subjects factor: Group [right-DBS, left-DBS, and controls]; within-subjects-repeated factor: Trial type [% correct no-stop trials, % correct go-only trials]). Again no statistically significant differences were found. We concluded that accuracies were very similar across both participants and experimental conditions.

**Table 6:** Results of the statistical analysis of the accuracy for right-DBS patients, left-DBS patients, and age-matched controls.

	**Value of parameters**	***p*-values**	**Effect size**	**BF_**10**_**
**Three-way ANOVA (factors: Group [right-DBS and left-DBS]; Trial type [Accuracy no-stop trials, Accuracy go-only trials]**				
**and DBS state [DBS-ON, DBS-OFF])**				
Main effect: Group	*F*_(1, 18)_ = 2.25	*p =* 0.16	$\eta_{\text{p}}^{2}$ = 0.11	0.97
Main effect: DBS state	*F*_(1, 18)_ = 2.66	*p =* 0.12	$\eta_{\text{p}}^{2}$ = 0.13	0.87
Main effect: Trial Type	*F*_(1, 18)_ = 0.45	*p =* 0.51	$\eta_{\text{p}}^{2}$ = 0.024	0.27
Interaction: Group*DBS state	*F*_(1, 18)_ = 0.04	*p =* 0.85	$\eta_{\text{p}}^{2}$ = 0.002	0.32
Interaction: Group*Trial Type	*F*_(1, 18)_ = 2.58	*p =* 0.13	$\eta_{\text{p}}^{2}$ = 0.13	0.75
Interaction: Trial Type*DBS state	*F*_(1, 18)_ = 2.97	*p =* 0.1	$\eta_{\text{p}}^{2}$ = 0.14	0.56
Interaction: Trial Type*DBS state*Group	*F*_(1, 18)_ = 0.006	*p =* 0.94	$\eta_{\text{p}}^{2}$ = 0.0003	0.38
**Two-way ANOVA (Group [right-DBS-ON, left-DBS-ON and Controls]; Trial type [Accuracy no-stop trials, Accuracy go-only trials])**				
Main effect: Group	*F*_(2, 39)_ = 0.54	*p =* 0.59	$\eta_{\text{p}}^{2}$ = 0.027	0.19
Main effect: Trial Type	*F*_(2, 39)_ = 0.72	*p =* 0.4	$\eta_{\text{p}}^{2}$ = 0.018	0.32
Interaction: Group*Trial Type	*F*_(2, 39)_ = 0.83	*p =* 0.44	$\eta_{\text{p}}^{2}$ = 0.041	0.27

*Task performance accuracy was defined as the ratio between the number of trials correctly executed and the total number of trials delivered, given by the sum of trials correctly executed, trials in which participants missed the target, trials in participants remained still on the central stimulus for more than 2 s, and trials in which they did not hold the central stimulus or the peripheral target for the requested amount of time. Bayes Factors report the ratio between the null vs. the alternative hypothesis (BF_10_); Partial eta-squared ($\eta_{p}^{\text{2}e}$). The suffix “DBS-ON” indicates that the DBS was in the ON state*.

## Discussion

### Inhibitory Control in Parkinson's Relays on the Activity of Both STN

As expected, we found that inhibitory control is impaired in Parkinson's patients with respect to healthy participants (11–13, 31). However, unilateral stimulation of STN does not improve either reactive or proactive inhibition, irrespective of whether the DBS was implanted in the right or in the left STN. This result is in full agreement with our previous works showing that only bilateral STN DBS restores a near-normal inhibitory control (12, 13). Although null results have to be interpreted with caution, here it is relevant to stress that on the one hand, the computation of Bayesian factors allow us to state that these findings are robust and that they are unlikely due to the variability of the sample or to statistical underpowering. On the other hand, the null effects could not be ascribed to the specifics of the task design, as in our previous works we gave the same task to STN DBS patients (12, 13). Even though it might be argued that previous studies are not fully comparable with the current one, as in the former cases patients were tested in OFF-drug-therapy, instead in the latter case they were tested in ON-drug-therapy in the current, we do not believe that this difference could affect the overall results for the two following reasons. First, there is evidence that dopaminergic treatment has an effect on inhibitory control just in early-stage but not in the moderate-to-advanced stages of PD (20), i.e., the clinical stages of all our patients. Second, a very recent finding showed that neither the unilateral stimulation of DBS contacts located in the ventral portion of STN nor the unilateral stimulation of DBS contacts located in the dorsal portion of STN affected inhibitory control in Parkinson's patients after 8 h of levodopa deprivation (32). These results are in full agreement with ours and were obtained in OFF-drug-therapy.

All in all, we confirm that the right STN does not play a key role in suppressing pending actions exploiting a different type of Parkinson's patient. However, as shown by several studies, the subthalamic nuclei are part of the brain network subserving inhibition (12–15, 33), but to implement this executive function both nuclei must be simultaneously active (12, 13, 15, 33). On this ground, it is plausible to suggest that inhibitory control might rely on cooperation between the two STN. More generally, it has been shown that the ability to cancel a pending movement is sustained by a bilateral brain network (8, 11, 34). Recently, Mirabella et al. (11) tested whether PD patients with a right onset of the disease, i.e., patients having a larger neurodegeneration of the left hemisphere, exhibit a better inhibitory control than PD patients with a left onset of the disease, as predicted by the hypothesis that suppression of a pending movement is computed by a right-lateralized frontal–basal ganglia–thalamic pathway (3). Mirabella et al. (11) found that both reactive and proactive inhibition were impaired in PD patients with respect to controls, but there were no differences between right- and left-onset PD patients. These results indicate that brain regions affected by PD play a key role in inhibition, but they also suggest that to achieve an efficient inhibitory control the two hemispheres must cooperate.

The discrepancy between the hypothesis of the lateralization of inhibitory control (3) and our findings pointing to the idea that this executive function relies on the cooperation between regions of the two hemispheres can be explained at least in two ways (11). The most trivial one is that the differences could be due to the type of movement that subjects have to cancel. In all our studies we required inhibition of arm-reaching movements (11–13) whereas usually subjects are required to inhibit key-press movements (16, 35–38). Arm-reaching movements are likely to require a different degree of control than key-press movements as the former are more complex and have a higher ecological relevance than the latter, being the only movements that allow physical interactions with the environment outside neurophysiology laboratories.

The second and, in our view, more likely explanation is given by the different task demands between study designs. Many of the experiments supporting the hypothesis of a right-lateralized inhibitory network exploited very demanding tasks in terms of attention or working memory loads such as the conditional stop-signal task (38). Others were relatively less cognitively demanding; however, they made use of centrally presented arrows (16). Such stimuli are known to induce endogenous shifts of attention toward the peripheral locations indicated by the arrows and as such they require top-down control (39). It has been shown that the same right-lateralized network is activated when tasks require either working memory maintenance (40), context monitoring (41) or endogenous attentional control (42). Therefore, it has been argued that in those instances the right-lateralized network supports attentional and working memory maintenance processes which are not specifically related to inhibitory control (42). In contrast, when, as in the case of the task we used, the go signal is given by the lighting of a peripheral stimulus, reflexive and automatic exogenous shifts of attention occur (43). As they require fewer resources, no clear signs of lateralization have been observed (8, 11–13, 44).

At this stage, one might wonder why the suppression of unilateral movements should involve a bilateral network. A possible answer comes from the increasing body of evidence indicating that, during the production of unilateral limb movements, both the contralateral and the ipsilateral motor cortices are activated (45–47). Even though the role of the ipsilateral activation is still controversial, it has been suggested that it reflects a functional inhibition of the motor cortex ipsilateral to the active hand, exerted by the contralateral hemisphere via the callosal fibers (46, 48). Given that the motor system is activated bilaterally when a unilateral arm movement is produced, it is plausible that also when it has to be suppressed both hemispheres must be involved. In addition, it has been reported that, to a certain extent, proximal muscles receive bilateral innervations from the dorsal pre-motor and the supplementary motor cortices (49). Likely, this pattern of innervation makes bilateral STN stimulation more effective than unilateral STN stimulation for proximal upper limb movements (50) as well as for their inhibition. As reaching arm movements involve both proximal and distal muscles it is plausible to suppose that bilateral STN DBS can allow a better motor control. Further studies will be needed to shed light on this issue.

### The Controversial Role of the DBS on STN

The STN plays a critical role in movements control by integrating cortical inputs from a wide array of areas and regulating the activity of the globus pallidus pars interna (GPi), the basal ganglia output nucleus which supervises arm movements (51). An increase in STN activity leads to an increase in the GPi discharge which, in turn, inhibits the motor thalamic nuclei, blocking their excitatory activity that typically precedes movement onset (51). This causes a decrease in the activity of the motor cortices, possibly slowing down or even suppressing the movement. Therefore, it has been suggested that the STN lies at the heart of the neural system controlling response inhibition (3, 12, 13) and conflict processing (52–54). In particular, it has been suggested that during conflict situations the activation of the STN raises the “decision threshold,” allowing generation of a temporary pause in motor output, leading to slower and more accurate responses (53). Local field potential (LFP) recordings from the STN are consistent with this hypothesis (55). In fact, on the one hand it has been shown that response inhibition is associated with specific changes in STN activity in several frequency bands (14, 56). On the other hand, it has also been shown that an increase in power of low-frequency bands occurs during conflict tasks (55, 57). This has been interpreted as the neural sign of delaying the decision-making during high conflict choices.

These two views of the role of STN are highly compatible. In fact, it might have been plausible to hypothesize that, in face of conflicts, STN might either slow down the motor response until enough evidence in favor of movement execution is collected, or it might cancel it if the planned movement is no longer valuable. However, results obtained from DBS behavioral studies provide discrepant results. Some research has shown that bilateral stimulation of STN induces impulsive responses in conflict conditions (53). Conversely, other evidence indicates that bilateral DBS of the STN markedly improves both reactive (13, 15, 33) and proactive inhibitory control in PD patients (12). In our view, the most likely explanation for this discrepancy lies in the different experimental contexts. Cognitive requirements to solve decision conflict tasks are rather different and often more complex than those required in the standard versions of the stop signal task, so the effect of DBS on the STN computations might produce different outcomes. Definitely, more studies are needed to clarify this issue.

### Unilateral vs. Bilateral DBS Implants: Clinical Relevance

Even though DBS of the STN markedly improves the motor symptoms of PD, the mechanisms underlying its effects remain to be clarified. A controversial clinical issue is whether to implant DBS unilaterally or bilaterally. This is because a number of studies have found that bilateral STN DBS may be associated with declines in executive function, such as verbal learning and memory (58, 59), or may even lead to adverse neuropsychological effects (19). Studies on comparative effects of unilateral and bilateral DBS of the STN have provided contrasting results. A recent study has shown that bilateral DBS improves the performance during dual-task conditions more than does unilateral DBS (60); however, under a different experimental setting it has previously shown the opposite (58). Lizarraga et al. (61) demonstrated that bilateral STN DBS yields greater improvement in motor performance than unilateral STN, even though DBS of the right STN seems to produce some benefits as well. Finally, Walker et al. (62) found no differences in weight changes following unilateral and staged bilateral STN. Our current results add a new piece of information to the debate as they significantly strengthened the idea that as far as inhibitory control is concerned the unilateral implant is not effective. A crucial step to better understand the risks and benefits of the bilateral approach would be to assess whether PD patients bearing unilateral DBS would have improved inhibitory control after the implant of the second DBS.

## Conclusions

In the current study, exploiting a different type of Parkinson's patient with respect to previous studies (unilateral vs. bilateral STN DBS patients), we confirmed that unilateral stimulation either of the right or of the left STN does not improve inhibitory control (12, 13). This finding indicates that the right STN does not play a key role in suppressing pending actions. Far from denying the role of STN in this executive function, we suggest that efficient inhibitory control is achieved only when the two STN are stimulated. Thanks to our approach, we can exclude that these results could be due to the variability of the sample or to statistical underpowering or that they could be explained by the specifics of the task design, given that in previous works we gave the same task to STN DBS patients (12, 13). The present findings, coupled with previous ones (12, 13), are of significance for understanding the effects of STN DBS on key executive functions, such as impulsivity and inhibition and they are also of clinical relevance for determining the therapeutic benefits of STN DBS as they indicate that, at least as far as inhibitory control is concerned, only bilateral STN DBS is effective.

## Author Contributions

CM collected data, made the figures, critically revised the manuscript. NM recruited patients, made the clinical survey, critically revised the manuscript. MS collected data, collaborated to localize DBS contacts, critically revised the manuscript. LP collaborated to localize DBS contacts, critically revised the manuscript. GG localized DBS contacts, critically revised the manuscript. RM performed surgery on patients, collaborated to localize DBS contacts, critically revised the manuscript. GM conceived and designed the experiments, programmed the temporal arrangements of stimulus presentation, analyzed the data, using self-made Matlab programs and IBM SPSS Statistics package; wrote the first draft of the manuscript.

### Conflict of Interest Statement

The authors declare that the research was conducted in the absence of any commercial or financial relationships that could be construed as a potential conflict of interest.

## Footnotes

^1^Reactive inhibition refers to the ability of a subject to react to the stop instruction, and it is measured by the reaction time to the stop signal or stop-signal reaction time.

^2^Proactive inhibition refers to the ability of subjects to shape their response strategy in anticipation of known task demand, which, in the case of the stop-signal task, is given by the awareness of the infrequent and unpredictable presentation of an imperative stop-signal.
